# The Progress and Evolving Trends in Nucleic-Acid-Based Therapies

**DOI:** 10.3390/biom15030376

**Published:** 2025-03-05

**Authors:** Yunlong Liu, Chunmiao Wang, Xiuping Fu, Mengtian Ren

**Affiliations:** School of Chemistry and School of Life Sciences, Tiangong University, Tianjin 300387, China; liuyunlong@tiangong.edu.cn (Y.L.); 2431161548@tiangong.edu.cn (C.W.)

**Keywords:** nucleic-acid-based therapeutics, nucleic acid drugs’ mechanisms, chemical modification, delivery systems, clinical use, trends and challenges

## Abstract

Nucleic-acid-based therapies have emerged as a pivotal domain within contemporary biomedical science, marked by significant advancements in recent years. These innovative treatments primarily operate through the precise binding of DNA or RNA molecules to discrete target genes, subsequently suppressing the expression of the target proteins. The spectrum of nucleic-acid-based therapies encompasses antisense oligonucleotides (ASOs), small interfering RNAs (siRNAs), microRNAs (miRNAs), and messenger RNAs (mRNAs), etc. Compared to more traditional medicinal approaches, nucleic-acid-based therapies stand out for their highly targeted action on specific genes, as well as their potential for chemical modification to improve resistance to nucleases, ensuring sustained therapeutic activity and mitigating immunogenicity concerns. Nevertheless, these molecules’ limited cellular permeability necessitates the deployment of delivery vectors to enhance their intracellular uptake and stability. As nucleic-acid-based therapies progressively display promising pharmacodynamic profiles, there has been a burgeoning interest in these treatments for applications in clinical research. This review aims to summarize the variety of nucleic acid drugs and their mechanisms, evaluate the present status in research and application, discourse on prospective trends, and potential challenges ahead. These innovative therapeutics are anticipated to assume a pivotal role in the management of a wide array of diseases.

## 1. Introduction

Nucleic-acid-based therapies (NABTs) represent a class of agents that directly target and regulate gene expression or functions at the molecular level. These drugs are designed to selectively suppress the production of disease-associated proteins, offering a precise and effective means of treating a wide range of genetic diseases, tumors, viral infections, and other conditions. The significance of nucleic acid drugs (NADs) lies in their ability to fundamentally alter the expression of disease-causing genes, which traditional small-molecule drugs and antibody drugs often cannot achieve. With advantages, such as a simple design, short research and development cycles, strong target specificity, extensive therapeutic areas, and long-lasting effects, NADs are poised to become the third major category of drugs following small-molecule drugs and antibody drugs [1,2,3].

In contrast to conventional antibody and small-molecule therapies, NADs boast a notable edge in targeting specificity [4]. By undergoing chemical modifications, nucleic-acid-based pharmaceuticals can be refined to further enhance their targeting accuracy. Studies have demonstrated that adjusting the chemical structure of phosphate groups, nucleobases, and ribose within nucleotides can significantly reduce the off-target effects observed in unmodified natural nucleic acids when tested in animal models. Moreover, the modified synthetic nucleic acids exhibit improved resistance to enzymatic degradation [5], increased lipophilicity, and decreased immunogenicity, ultimately leading to an enhancements in the drug’s effectiveness and efficiency.

The selection of an appropriate delivery vector and the innovation of effective delivery mechanisms are vital to the advancement of NAD research. The large molecular size and strong negative charge of NADs hinder their ability to penetrate cells, resulting in poor cellular uptake. The development of delivery systems has addressed this challenge to some extent, particularly in enhancing the therapeutic efficacy of NADs in vitro. However, the efficient and widespread tissue delivery of oligonucleotide therapeutics faces challenges due to their large size, polyanionic nature, and susceptibility to degradation, requiring them to withstand extracellular nucleases, evade renal excretion, and bypass clearance mechanisms while successfully traversing the capillary endothelium and reaching precise intracellular targets. Current strategies primarily focus on local delivery to immune-privileged sites, like the eye or liver, with systemic delivery to the CNS posing additional hurdles due to the blood–brain barrier, although the direct injection into cerebrospinal fluid shows promise [6]. Despite progress in local delivery, developing effective technologies for extrahepatic systemic delivery remains a key objective, with the liver being a preferred target due to its rapid uptake of oligonucleotides and high receptor concentrations, particularly the asialoglycoprotein receptor, which specifically targets GalNAc conjugates, while other highly vascularized tissues, like the kidneys and spleen, also accumulate oligonucleotides [7]. The synergy of chemical modifications with sophisticated delivery systems addresses the inherent limitations of NADs, conferring clear benefits in terms of heightened specificity, efficiency, and prolonged activity [8], marking a significant milestone in the realm of NAD research.

Common NADs employed in gene therapy include antisense oligonucleotides, small interfering RNA (siRNA), aptamers, and messenger RNA (mRNA), among others. The mechanisms of action for these various drugs differ. For example, antisense oligonucleotides and siRNA disrupt target gene expression by engaging in Watson–Crick base pairing with pre-mRNA or mRNA [9], leading to therapeutic benefits; mRNA can directly induce the production of target proteins within cells and facilitate gene expression, regulating the expression levels of proteins [10], thus fulfilling the drug’s therapeutic role. Additionally, aptamer RNA can bind to disease-associated proteins with its distinctive three-dimensional structure, thereby precisely modulating the functional interactions of these proteins.

**Figure 1:**
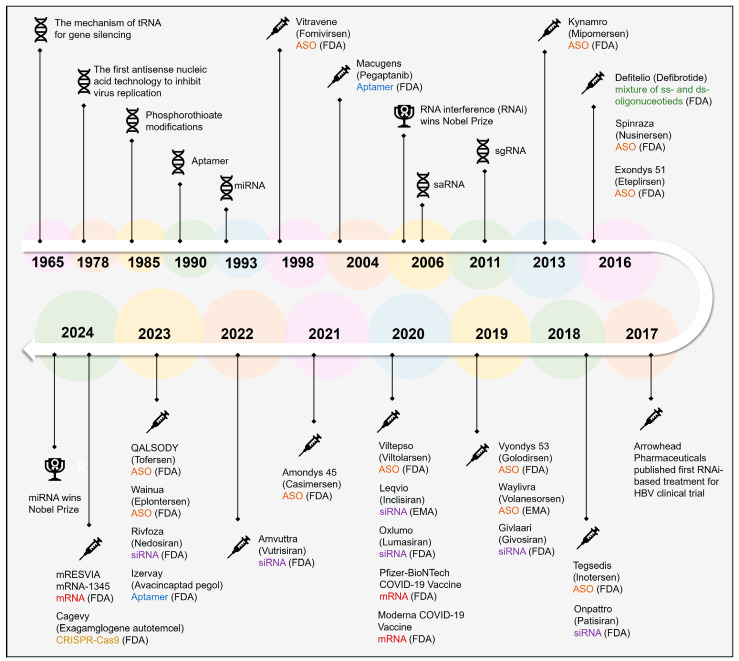
The pivotal evolutionary journey of NADs [8,11,14,17,18,19,20,21,22,23,24,25,26,27,28,29,30,31,32,33,34,35,36,37,38,39,40,41,42,43,44,45].

The potential of NABTs has been unfolding since the latter half of the 20th century. In 1965, investigators identified the reactivity of antisera to yeast small RNA (sRNA) [11], which subsequently catalyzed the progressive development of research focused on the utilization of small-RNA-based therapeutics. In 1978, researchers found that antisense oligonucleotides (ASOs) could bind to mRNA through base-pair complementarity, surprisingly, inhibiting the production of specific proteins encoded by the mRNA [12,13]. In 1998, the U.S. Food and Drug Administration (FDA) approved the first ASO drug, formivisen [14], for treating cytomegalovirus-induced retinitis, heralding the dawn of nucleic-acid-based medications in the 21st century. The following year, in 1998, Fire [15] and Mello identified double-stranded RNA molecules capable of inducing the RNA interference effect in Caenorhabditis elegans, which they termed small interfering RNA (siRNA). It was not until 2018 that patisiran [16], a siRNA drug for treating hereditary transthyretin amyloidosis-related peripheral neuropathy, received approval from the FDA, establishing a solid foundation for the research on siRNA-based pharmaceuticals. Figure 1 compiles the pivotal milestones in the evolution of NADs, offering a concise overview of their developmental trajectory.

To this day, about 19 NADs have gained approval from the FDA or the European Medicines Agency (EMA). These drugs have demonstrated utility in the treatment of a range of conditions, including genetic disorders, cancer, and hepatitis, among others. In the current century, a growing number of biotech companies are investing in the research and development of NABTs, fueling a thriving sector in the pharmaceutical industry.

## 2. Chemical Modifications of Nucleic-Acid-Based Therapeutics

Chemical modification represents a significant approach for improving the delivery of nucleic acid therapeutics and has seen extensive applications in recent years. In comparison to unmodified nucleic acid drugs, modified variants demonstrate enhanced resistance to enzymatic degradation and a heightened affinity [46] for target molecules. Such chemical modifications facilitate the increased efficacy of nucleic acid drugs in the treatment of a range of diseases. Presently, prevalent modification strategies [47] encompass alterations to the phosphate backbone, modifications to the ribose sugar, base modifications, and various other specific modifications.

### 2.1. Phosphate Backbone Modifications

**Figure 2:**
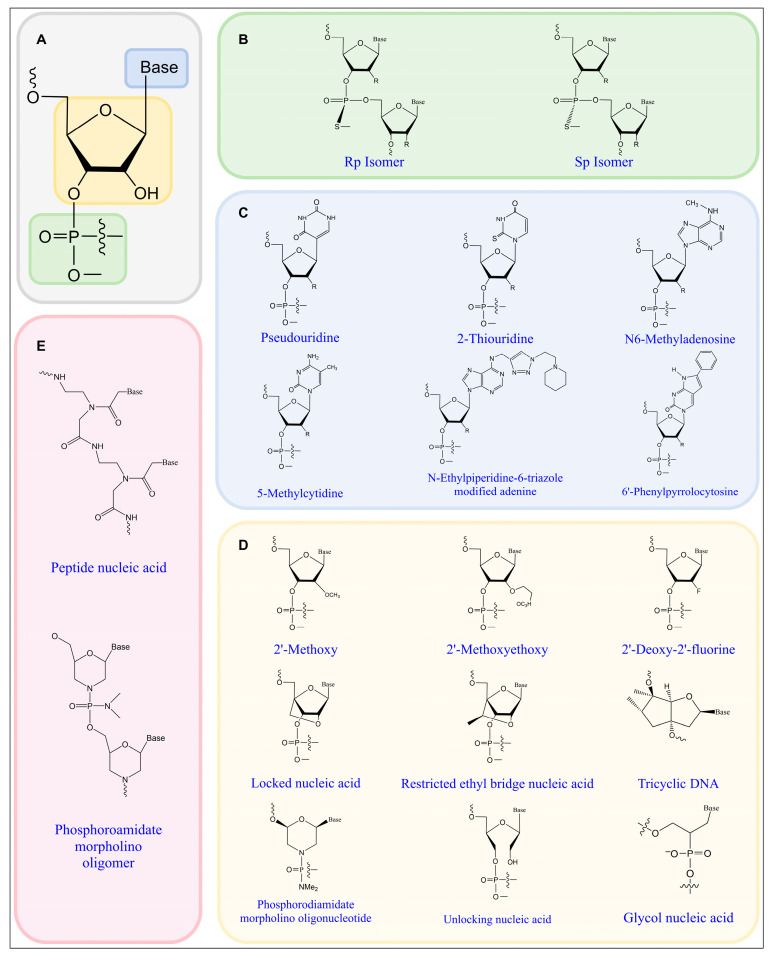
Different chemical modifications of nucleic-acid-based therapeutics. (**A**) Structural unit of natural ribonucleotide. (**B**) Rp and Sp structure of thiophosphate ester. (**C**) Structure of several base modifications. (**D**) Structure of several ribose modifications. (**E**) Advanced modifications.

The alteration of the phosphate backbone represents a prevalent approach in the contemporary domain of nucleic-acid-based drug development and applications. Among the various modifications, the phosphorothioate (PS) [48] modification is characterized by the substitution of the unbridged oxygen atom in the phosphate group with a sulfur atom, resulting in the formation of a phosphorothioate bond. This modification significantly enhances the resistance of nucleic acids to nucleases, thereby prolonging their half-life in vivo. Furthermore, it markedly improves the binding affinity [49] of the modified nucleic acids to plasma proteins, which contributes to an increased persistence of the nucleic acid drug within the bloodstream and enhances the probability of interactions with target cells, ultimately improving the drug’s bioavailability. It is important to note that the modified phosphate backbone can exist in both the Sp and Rp conformations (Figure 2B). Iwamoto [50] and his research team have shown that the stereoisomerization of phosphorothioates has a substantial impact on the therapeutic effectiveness of antisense oligonucleotides (ASOs). Of the two stereoisomeric forms, the Sp conformation demonstrates superior stability compared to the Rp conformation. This enhanced stability allows ASOs that incorporate the Sp conformation of phosphorothioate to achieve prolonged inhibition in vivo, thereby extending the duration of their effective action.

Fomivirsen [51], an antisense oligonucleotide drug that has received marketing approval, is utilized in the treatment of AIDS-associated cytomegalovirus retinitis. The phosphorothioate modification incorporated in this drug allows it to maintain sufficient stability following intravitreal injection, effectively inhibiting viral gene expression.

### 2.2. Base Modifications

Base modifications represent a significant component of nucleic acid alterations, primarily encompassing base substitutions and the modification of specific nucleobase positions, notably the 5-position of pyrimidines and the 8-position of purines. Notable examples of base modifications include pseudouridine and 2-thiouridine [52], among others. (Figure 2C).

Pseudouridine, in particular, is commonly employed as a substitute for uracil. Research conducted by Vaidyanathan [53] et al. has demonstrated that this modification enhances the translation efficiency of mRNA encoding the Cas9 protein while simultaneously reducing its immunogenicity. Furthermore, N-ethylpiperidine-6-triazole modified adenosine analogs have been shown to decrease immunogenicity by inhibiting the interaction between small interfering RNAs (siRNAs) and Toll-like receptor 8 [54]. The 6′-phenylpyrrole cytosine [55], a cytosine analog, exhibits superior base pairing capabilities, thermal stability, and pronounced fluorescence. Notably, modifications to siRNA do not diminish the gene-silencing efficacy; rather, their fluorescent characteristics facilitate the monitoring of siRNA uptake within cells. Following the modification of siRNA, the gene-silencing effect remains unaffected, and the fluorescence can be employed to trace the uptake and intracellular transport of siRNA, thereby aiding in the exploration of its intracellular mechanisms.

### 2.3. Ribose Modifications

Ribose modifications are predominantly classified into two categories. The first category encompasses the addition of diverse groups of varying sizes and polarities at the 2′ position of ribose, which includes modifications, such as 2′-methoxy, 2′-methoxyethoxy, and 2′-deoxy-2′-fluoro [56], among others. The second category involves concurrent modifications at the 2′ position along with other sites on the ribose molecule, as exemplified by locked nucleic acid (LNA), as depicted in Figure 2D.

Modifications at the 2′ position are essential for augmenting the resistance of nucleic acids to nuclease-mediated hydrolysis. The 2′-methoxy modification is particularly prevalent due to its ability to enhance the binding affinity of the drug to target mRNA, inhibit nuclease hydrolysis [57], and diminish the immunogenicity of the nucleic acid in vivo. Additionally, this modification contributes to a degree of lipid solubility, thereby improving the pharmacokinetic profile of the nucleic acid. Its analog, 2′-methoxyethoxy, shares a similar chemical structure but demonstrates a greater affinity for target mRNA and enhanced resistance to enzymatic degradation. Moreover, the 2′-deoxy-2′-fluoro modification can significantly bolster the affinity and stability of small interfering RNA (siRNA) towards its target.

Furthermore, modifications at the 2′ and 4′ positions of the ribose, or even alterations to the entire sugar ring, have proven to be effective. Locked ribonucleic acid (LNA) adopts a C3′-endo conformation, which enables the short sequence to maintain a high affinity for its target while exhibiting resistance to enzymatic degradation. However, this short sequence may also lead to off-target effects and toxicity concerns. Consequently, it is often employed in place of modifications associated with unlocked ribonucleic acid (UNA) and other variants. The integration of these four ribose modifications with phosphorothioate modifications has been shown to enhance therapeutic efficacy.

### 2.4. Other Specialized Modifications

Peptide nucleic acid (PNA) is a synthetic analog of nucleic acids distinguished by its neutral peptide backbone, specifically an amide bond formed with 2-aminoethylglycine, which substitutes the traditional pentose phosphodiester bond. This structural alteration significantly enhances PNA’s resistance to hydrolysis by nucleases and proteases, thereby increasing its stability in both in vivo and ex vivo environments. As a result, PNA can remain within biological systems for prolonged durations, functioning effectively within the framework of phosphoramidite morpholino oligomers (PMOs). PMO is charge-neutral within the physiological pH range due to the linkage of a phosphoramidite group and a phosphorodiamide group. This neutrality reduces the likelihood of ionization, thereby enhancing stability and the affinity for target molecules. Further modifications, such as the addition of membrane-penetrating peptides [58] and positively charged amine groups, can augment transmembrane transport capabilities, thus improving the efficacy of drug delivery into cells.

## 3. Delivery Strategies for Nucleic Acid Drugs

**Figure 3:**
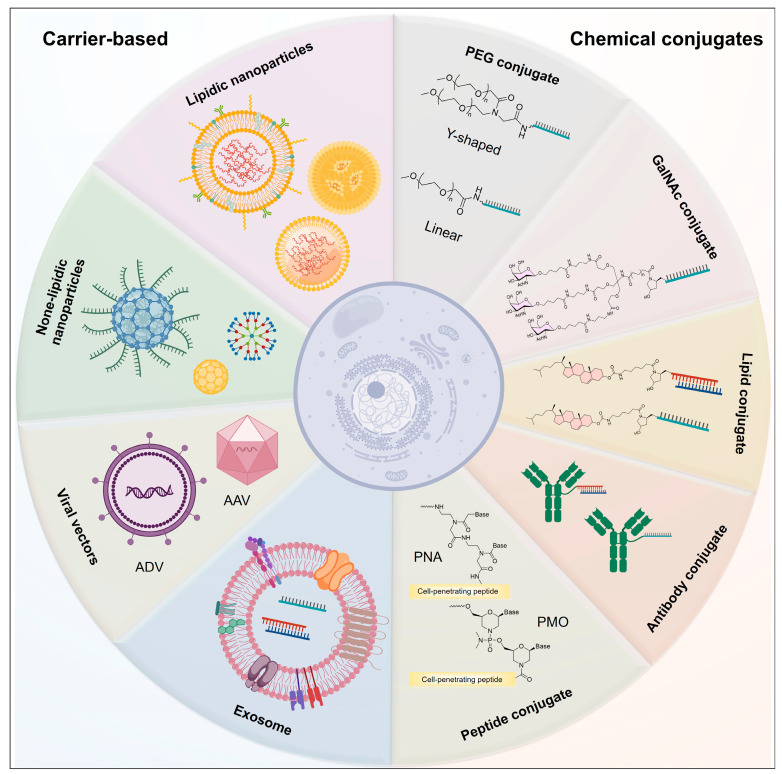
Delivery strategies of nucleic acid drugs.

Chemical-modification strategies can enhance the stability of nucleic-acid-based drugs, improve their affinity for target molecules, and optimize their delivery efficiency to a certain extent. However, due to their large size, negative charge, and hydrophilic nature, nucleic-acid-based drugs exhibit poor cellular permeability, making it difficult for them to cross the cell membrane and enter the cell interior, thereby hindering their effectiveness in vivo. Here, we will systematically review and delve into the research progress and development trends of various delivery strategies in the field of nucleic acid drug (Figure 3) [59,60].

### 3.1. PEG Conjugates

Polyethylene glycol (PEG) is a highly adaptable, non-ionic, and hydrophilic polymer featuring functionalizable termini. PEG conjugates can prolong the renal clearance of nucleic acid drugs, thereby extending the drug residence time in the body. Pegaptanib, a targeted anti-vascular endothelial growth factor (VEGF) aptamer drug for ocular vascular diseases, comprises 28 nucleotides coupled with a 40 kDa PEG moiety [61,62]. Additionally, the process of PEGylation prolongs the circulation time of drugs by minimizing renal excretion and bolstering the stability of nucleic-acid-based medications. The metabolic and toxicological profiles of PEGylated compounds hinge on the physicochemical attributes of the PEG segment, encompassing its molecular weight, the nature of terminal modifications, and the distinct configurations of PEG (either linear or branched).

### 3.2. Lipid-Conjugated Oligonucleotide

Cholesterol-functionalized siRNA stands as one of the pioneering lipid conjugates described in early studies. Through the direct modulation of an siRNA’s hydrophobicity, lipid conjugation aids in the specific incorporation into endogenous lipoprotein pathways, thus managing its pharmacokinetic characteristics [63]. Such a modification boosts the efficacy of siRNA-induced mRNA silencing particularly in tissues with a high density of lipoprotein receptors, encompassing the liver, adrenal glands, ovaries, and kidneys. Furthermore, the binding affinity of ASO–fatty acid conjugates to plasma proteins increases with the length of the fatty acid chain [64]. Research by Wang et al. has shown that lipid conjugates, including free fatty acids, tocopherols, and cholesterol, enhance the binding of proteins to PS-ASO and increase membrane association and intracellular uptake [65].

### 3.3. GalNAc Conjugates

The trimer of N-acetylgalactosamine (GalNAc) group is one of the most successful tissue-targeting ligands. GalNAc exhibits a strong binding capability to the asialoglycoprotein receptor (ASGPR), a receptor involved in endocytosis that is abundantly present on hepatocyte membranes. This interaction aids in the efficient uptake of GalNAc-linked nucleic acid medications from the hepatocyte surface into the cytoplasmic compartment [66]. Subsequently, the GalNAc moiety undergoes enzymatic degradation, releasing the oligonucleotide to initiate gene-regulating activity [67,68]. It is worth noting that during delivery, the nucleic acid molecules within these conjugates are directly in contact with serum, making it a significant challenge to bolster their stability within physiological settings. The pharmacokinetics (PK) and pharmacodynamics (PD) of GalNAc-conjugated oligonucleotides have been extensively evaluated in animals and humans [69,70]. Alnylam Pharmaceuticals has extensively explored the conjugation of GalNAc with siRNA for liver-specific delivery. Givosiran, developed by Alnylam, targets 5′-aminolevulinate synthase 1 (ALAS1) for the treatment of acute hepatic porphyria and received FDA approval in November 2019 [29]. Inclisiran, the second GalNAc-conjugated siRNA, featuring 2ʹ-F, 2′-OMe, and PS modifications, targets PCSK9 in the liver to reduce LDL cholesterol levels and exert lipid-lowering effects [34]. In 2023, the FDA approved nedosiran, a tetravalent covalently linked GalNAc-siRNA targeting the silencing of lactate dehydrogenase A (LDHA) in hepatocytes [41].

### 3.4. Antibody-Conjugated Oligonucleotide

Antibodies represent a highly promising form of a carrier delivery system, capable of serving either as direct conjugates to enhance the delivery and targeting capabilities of nucleic acid drugs or as surface modifiers for non-conjugated carriers, such as liposomes. However, direct conjugation technology is still in its early stages of research. MXD3 (MAX dimerization protein 3) is involved in cell proliferation and functions as an anti-apoptotic protein, while CD22 serves as a critical surface protein that regulates B cell receptor signaling. Satake et al. innovatively developed conjugates of MXD3 antisense oligonucleotides (ASOs) with anti-CD22 antibodies, achieving the precise delivery of ASOs to leukemic target cells [71] Another study focused on antigens expressed by glioblastoma stem cells (GSCs), such as CD44 and EphA2, conjugating antibodies targeting these antigens with chemically modified ASOs designed for renal cell carcinoma. These ASOs utilized a hybrid approach, integrating DNA with 2′-deoxy-2′-fluoro-β-D-arabinonucleic acid (FANA), to boost their resistance to nucleases and enhance their affinity for mRNA [72]. Experimental results demonstrated that these antibody-conjugated ASOs effectively reduced the phenotypes of patient-derived GSCs. Additionally, the Huda research group utilized monoclonal antibody (mAb)–small interfering RNA (siRNA) conjugates targeting the B cell activating factor receptor (BAFF receptor) or B cell maturation antigen (BCMA) receptor, successfully improving clinical symptoms in patients with myasthenia gravis (MG) [73,74]. Another study employed siRNA conjugated with anti-CD71 Fab targeting hypoxanthine-guanine phosphoribosyltransferase (HPRT), for targeting skeletal and cardiac muscle tissues. This conjugate elicited potent and sustained silencing effects in muscular organs, holding promise for the treatment of diseases, such as myotonic dystrophy and Duchenne muscular dystrophy [75].

### 3.5. Peptide Conjugates

The peptide is a highly promising coupling form for nucleic acid drug delivery, capable of endowing therapeutic oligonucleotide conjugates with cell-penetrating properties (i.e., CPP), tissue/cell targeting, or improved endosomal escape. Due to the aggregation caused by electrostatic interactions between negatively charged nucleic acid drugs and positively charged CPPs, CPPs typically restrict their covalent coupling to charge-neutral nucleic acid backbones, such as PNA and PMO [76,77].

Cell-penetrating peptides (CPPs) are short peptides composed of 5-30 amino acids, which can directly cross the cell membrane or be taken up by cells through endocytosis. Therefore, they enhance the intracellular delivery of nucleic acid drugs, thereby achieving targeted therapeutic goals. Despite their therapeutic potential, CPPs lack selectivity and face challenges in stability and biological activity during delivery. Modifying the termini of existing CPPs or adjusting the peptide backbone (e.g., through peptide cyclization like bivalent cRGD) can enhance their stability and cellular permeability [78]. However, CPPs usually require high concentrations and doses to achieve therapeutic effects, leading to significant toxicity and side effects, thereby limiting their clinical application. Recent studies suggest that promoting the formation of CPP multimers and employing multi-functional peptide conjugates can somewhat enhance delivery effectiveness [79]. Studies have shown that bivalent cRGD successfully transports VEGFR2-siRNA into tumor cells reducing cancer progression [78].

### 3.6. Lipidic Nanoparticles

Lipidic nanoparticles have emerged as a highly promising technology for the delivery of nucleic acid drugs, offering significant advancements in efficiency and safety. In terms of their classification, LNPs include liposomes, which have a hydrophilic core and a lipid bilayer shell, and can encapsulate hydrophilic drugs, like nucleic acids [80]. Lipid nanoparticles (LNPs) differ from liposomes by lacking a closed lipid bilayer structure and internal aqueous core and can be composed of various lipids, including cholesterol, helper lipids, and PEGylated lipids. Lipid nano-emulsions (LNEs) are nanodroplets stabilized by phospholipids and emulsifiers, while solid lipid nanoparticles (SLNs) have a surfactant monolayer shell and a solid lipid core. Nanostructured lipid carriers (NLCs) are hybrid formulations between LNEs and SLNs, with cores composed of both liquid oil and solid lipid phases. The principles of nucleic acid delivery via LNPs involve the use of self-assembled nanostructures with a diameter of approximately 100 nm that can bind to negatively charged nucleic acids through electrostatic interactions [80,81]. These nanoparticles encapsulate the nucleic acid drugs in their cores to protect them from nuclease degradation during delivery. Classical LNPs comprise ionizable lipids (ILs) or cationic lipids (CLs), cholesterol, helper lipids, and lipids modified with polyethylene glycol (PEG) [82].

LNPs have been extensively used in the delivery of various nucleic acid drugs, such as antisense oligonucleotides (ASOs), small interfering RNAs (siRNAs), and messenger RNAs (mRNAs). LNPs technology is currently one of the most widely used applications for the delivery of nucleic acid drugs. Notable examples include patisiran and onpattro, both FDA-approved siRNA drugs, and COVID-19 mRNA vaccines, like BNT162b2 (Comirnaty) and mRNA-1273 (Spikevax) [83,84,85]. Despite their advantages, LNPs face challenges, such as PEG antibody formation and reduced liver uptake, which researchers are addressing by optimizing LNP compositions and developing new strategies, such as introducing selective organ-targeting components. In conclusion, LNPs have revolutionized the delivery of nucleic acid drugs, and by understanding their classification, principles, applications, and challenges, researchers can continue to innovate and improve this technology, leading to more effective and widely accessible treatments for patients.

### 3.7. None-Lipidic Nanoparticles

Non-lipid nanoparticle delivery strategies, primarily comprising cationic polymer nanoparticles (CPNs) and inorganic nanoparticles (INPs), have emerged as research hotspots in the field of nucleic acid drug delivery. CPNs form complex structures with nucleic acid drugs through electrostatic interactions, with representative polymers, such as polyethyleneimine (PEI), polyamidoamine dendrimers (PAMAM), and poly-L-lysine (PLL), exhibiting high efficiency in gene transfection but also posing challenges in terms of cytotoxicity and biodegradability [86,87]. To address these limitations, researchers have enhanced the transfection efficiency and reduced toxicity through functional modifications [88,89,90]. Meanwhile, inorganic nanoparticles, including gold nanoparticles (AuNPs), silicon dioxide nanoparticles (SiNPs), magnetic nanoparticles (MNPs), and carbon nanotubes (CNTs), have demonstrated significant potential in nucleic acid drug delivery due to their stable structures, large specific surface areas, and adjustable surface properties [91]. Notably, studies have confirmed the feasibility of AuNPs and SiNPs in drug–siRNA co-delivery and targeted gene delivery for cancer treatment [92,93].

The innovation of non-lipid nanoparticle delivery strategies in nucleic acid drug delivery lies not only in the selection of materials but also in the functional modification of materials and the investigation of delivery mechanisms. For instance, strategies, such as coating with hyaluronic acid shells, have successfully reduced the cytotoxicity of PLL nanoparticles and enhanced Green Fluorescent Protein (GFP) gene expression in HEK-293T cells [90]. Furthermore, the development of AuNP-mediated drug delivery platforms and spherical nucleic acid drugs, like NU-0129, has provided new therapeutic strategies for refractory diseases, such as glioblastoma multiforme (GBM) [92]. Additionally, the superparamagnetic properties and site-specific targeting capabilities of MNP, as well as the chemical modification and drug-loading capacity of CNT, offer possibilities for the widespread application of non-lipid nanoparticles in nucleic acid drug delivery [94,95]. In summary, non-lipid nanoparticle delivery strategies exhibit diversity and flexibility in nucleic acid drug delivery, offering new avenues for disease treatment and holding broad application prospects.

### 3.8. Exosomes

Exosomes are membrane vesicles ranging from 40 to 160 nm in diameter, capable of serving as natural nanocarriers for loading a variety of drugs, particularly nucleic-acid-based drugs, such as CRISPR-Cas9, ASO, siRNA, miRNA, and mRNA [96]. Equipped with transmembrane- and membrane-anchored proteins, exosomes enhance endocytosis, facilitating drug delivery [97]. The CD47 molecules on their surface enable escape from phagocytosis, thereby improving drug stability [98]. Exosomes exhibit low immunogenicity, high biocompatibility, and safety, and possess the ability to cross biological barriers. Exosomes derived from different sources exhibit distinct functions; for instance, those from human embryonic kidney HEK293 cells are immunologically inert, cancer-cell-derived exosomes can be used for targeted delivery, and immune-cell-derived exosomes can evade immune phagocytosis [99]. Studies have utilized exosomes for delivering nucleic acid drugs to treat cancer. For example, Kim et al. employed ovarian-cancer-derived exosomes to deliver CRISPR-Cas9 plasmids, inhibiting tumor growth in SKOV3 xenograft mice [100]. Other studies have also demonstrated the favorable antitumor effects of exosome-delivered nucleic acid drugs [96]. However, exosome-based drug delivery faces challenges, such as large-scale production, the development of novel drug-loading methods, and the influence of the internal environment. Yang et al. reported a cellular-nanoporation method for high-yield exosome production [101], while Hu et al. utilized LNP-like structures to load siRNA into exosomes [102]. Exosomes produced by tumor cells from patients themselves can be harnessed for targeted delivery [103]. When exosomes are used as carriers for nucleic acid drug delivery, efficient drug-loading strategies must be considered, including exogenous pathways that may compromise exosome integrity and endogenous pathways that require further research. The lipids and proteins on the exosome surface provide binding sites for targeted ligands, which can be covalently attached to enhance targeting capabilities, as exemplified by T7-peptide-modified exosomes delivering AMO-21 to glioblastoma cells [104]. Exosomes can also be genetically engineered to express ligands, such as LAMP-2B gene modification for targeted delivery [105]. Approximately 40 companies worldwide have developed exosome-based therapies, but challenges, such as large-scale production, purity, and batch uniformity, limit their clinical application [106]. The absence of regulatory standards for exosome-based therapeutic drugs, taking into account safety, effectiveness, and quality control, highlights the pressing requirement for standardized approaches and guidelines to oversee these molecules.

## 4. Antisense Oligonucleotides (ASOs)

**Figure 4:**
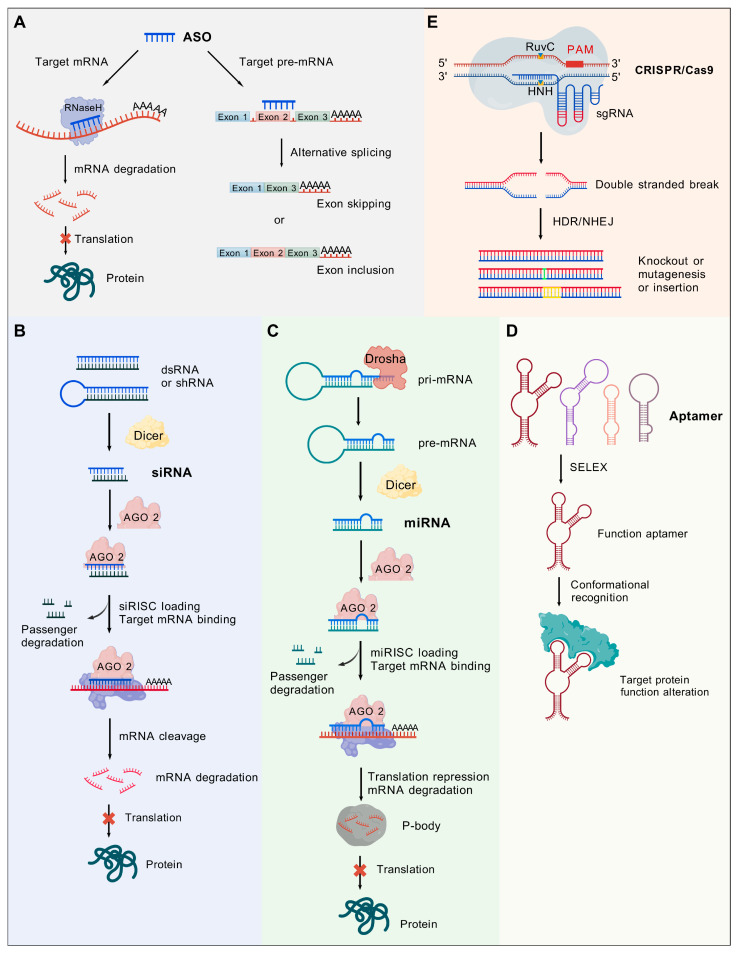
Elucidating the mechanistic underpinnings of RNAi and showcasing exemplary NABTs. (**A**) ASO. (**B**) siRNA. (**C**) miRNA. (**D**) Aptamer. (**E**) CRISPR-Cas9. Created with BioGDP.com [122].

An ASO is a synthetic single-stranded or double-stranded oligonucleotide typically ranging from 12 to 30 nucleotides in length [107]. They function by binding to target mRNA sequences through Watson–Crick base-pairing rules [108], resulting in the formation of an ASO–RNA double-stranded complex. The mechanisms by which ASOs operate are two-fold (Figure 4A): (1) RNase-H-dependent mechanism, within the cell nucleus, ASOs (containing at least partial natural DNA sequences) selectively bind to target RNA, forming DNA–RNA heteroduplexes. This structure activates RNase H endonuclease, which rapidly cleaves the RNA strand of the hybrid, leading to degradation of the target RNA and subsequent reduction of its levels [109,110]. (2) RNase H-independent mechanism: This approach involves modifying and altering the target RNA. Certain ASOs possess a distinctive three-dimensional structure that, when bound to mRNA, may not result in cleavage but can destabilize the mRNA [111]. Moreover, by engaging in specific base pairing, ASOs can bind to pre-mRNA sequences, either blocking or promoting specific splicing events, like exon skipping, intron retention, or alternative splicing. Consequently, this interaction alters the sequence of mature mRNA and impacts protein synthesis, revealing a promising regulatory mechanism for the treatment of numerous inherited metabolic diseases [112]. Consequently, antisense oligonucleotides offer a highly targeted approach to gene expression regulation through these dual mechanisms [110].

ASOs are anionic compounds and are primarily taken up by cells through endocytosis. To observe the effects of ASOs on target genes within cells, they must reach a certain concentration within the cells. However, the permeability of most cells is poor, making it difficult for ASOs to accumulate to high concentrations within the cells. To address this issue, researchers have developed various delivery systems to enhance cellular permeability, which is conducive to the accumulation of ASO concentrations. Encapsulating ASOs in lipid nanoparticles (LNPs) is the most advanced method for delivery at present [113], as LNPs have abilities to efficiently and targetedly deliver drugs to target cells, while simultaneously protecting the drugs from degradation and exhibiting excellent biocompatibility and potential for multifunctionalization. Typical strategies involve conjugating LNPs with antibodies (immuno-liposomes) to achieve effective and specific targeting [114]. For instance, Sicard et al. successfully targeted prostate cancer cells expressing Her-2 by encapsulating Her-2 antibody-conjugated ASOs within liposomes (termed ASO-iLi). Their research demonstrated that ASO-iLi exhibited greater efficacy than both unconjugated free ASOs and liposomes encapsulating ASOs without antibody conjugation [115]. Furthermore, a novel strategy employs stimuli-responsive liposomes that release their contents in response to specific stimuli, such as pH levels, the redox status, the temperature, and magnetic fields [116,117]. Upon endocytosis, the acidic environment of the endosome triggers the fusion of pH-responsive liposomes with the endosomal membrane, thereby facilitating the release of the cargo into the cytoplasm. This approach further enhances the delivery efficiency and specificity of ASOs encapsulated in LNPs.

In recent years, ASO drugs have developed rapidly, with many ASO drugs having entered clinical stages and demonstrated their efficacy. Studies by Wong et al. reveal that mipomersen (Kynamro, Seattle, WA, USA) [23] can exert its effects by binding to apoB-mRNA, playing a crucial role in the treatment of atherosclerosis and other diseases [118]. Meanwhile, Keam et al. found that inotersen (Tegsedi, Reading, UK) [28] can target the 3′ untranslated region (UTR) of the thyroid hormone transporter mRNA and induce its degradation, which is used for the treatment of hereditary transthyretin amyloidosis-related polyneuropathy. In addition, the FDA approved tofersen (BIIB067) in April 2023 for the treatment of ALS caused by heterozygous SOD1 variants [119]. While there are some limitations in clinical trials and reports of adverse events, its approval signifies a significant step forward in ALS therapy. By the end of 2024, a total of eleven ASO drugs had been approved and launched on the market (Figure 1). Compared to other types of drugs, ASOs have the most approved varieties, indicating that ASO drugs have a vast potential for development [120].

Antisense oligonucleotides (ASOs) present considerable benefits in the treatment of diseases, attributable to their capacity to selectively target specific mRNA sequences, effectively modulate gene expression, and exhibit high efficiency in research and development. Additionally, ASOs demonstrate improved stability and cellular uptake as a result of chemical modifications. Nonetheless, ASOs are also associated with several limitations, including suboptimal uptake efficiency due to the barriers posed by cell membranes, vulnerability to degradation in vivo, a relatively short half-life, the potential for eliciting immune responses, off-target effects, and elevated production costs [121].

In reviewing the current landscape of ASO therapy, it is evident that one of the primary limitations lies in the restricted delivery options to the affected tissues, posing significant challenges in achieving effective targeting. Additionally, there exists a notable gap in our understanding of the precise modulation levels of specific proteins required for therapeutic success across various diseases. Future research should focus on developing more sophisticated delivery systems and enhancing our knowledge of protein modulation to fully harness the potential of ASO therapy in treating a broader range of diseases.

## 5. Small Interfering RNAs (siRNAs)

RNA interference can occur through two mechanisms: the siRNA mechanism and the miRNA mechanism [123] (Figure 4B). Small interfering RNA (siRNA), also known as silencing RNA or short interfering RNA, refers to a dsRNA with a length of approximately 21-23 nucleotides. siRNA can bind to the Argonaute2 (AGO) proteinase in the cytoplasm, forming an RNA-mediated silencing complex, which is known as the RNA-induced silencing complex (RISC) [124]. RISC can precisely target mRNA and bind to it at a specific site with complete complementarity [125], effectively triggering mechanisms for cleavage or inhibiting translation. With ATP providing the energy for the entire process, RISC can separate double-stranded siRNA into single strands and tightly bind to them, forming a highly active RISC complex. The activated RISC can “lock” onto the target mRNA and cleave it. Furthermore, nucleases can catalyze the precise cleavage of the target mRNA into small fragments, which are gradually degraded, ultimately achieving the silencing of a specific gene.

The functional principle of therapeutic siRNAs mirrors that of biological siRNAs, yet therapeutic siRNAs exhibit a higher degree of specificity and targeting precision. These synthetic therapeutic siRNAs are delivered into cells through specialized delivery systems. Upon cellular entry, they specifically bind to their target mRNAs and induce the RNA-induced silencing complex (RISC) to cleave the target mRNA or suppress its translation. In the application process, siRNA necessitates enhancements across several fronts. Its rod-shaped, helical structure is relatively rigid, and the majority of siRNAs exhibit high negative charges, which contribute to their immunogenicity and instability. Researchers have addressed these issues by chemically modifying siRNA to reduce its immunogenicity and enhance stability. The degradation of siRNA is primarily mediated by the exonuclease Eri-1 at the 3′-phosphodiester bond [126], and thus, chemical modifications at this site can enhance Eri-1′s resistance to enzymatic degradation, making it a focal point in recent research.

Despite the improvements brought about by chemical modification strategies, the challenge of delivery remains a significant barrier to the robust, safe, and efficient application of siRNA drugs. Liposomes represent an appealing non-viral carrier for siRNA delivery [127]. siRNA can be encapsulated within lipid complexes by forming electrostatic interactions with positively charged liposomes [128]. Lipid nanoparticles (LNPs), alternatively termed stable nucleic acid lipid particles, constitute liposomes that incorporate ionizable lipids, phosphatidylcholine, cholesterol, and PEG-lipid conjugates in precise proportions [129,130], and they have demonstrated remarkable success across various applications. Notable milestones include the silencing of the hepatitis B virus and APOB [131] via siRNAs in preclinical animal studies, as well as the recent approval of patisiran [132]—an siRNA administered in an LNP formulation. The encapsulation of nucleic acid cargos within these LNPs offers robust protection against nuclease degradation both in circulation and within endosomes. Furthermore, ionizable LNPs exhibit affinity for APOE, which enhances their hepatic uptake through LDLR-mediated endocytosis. Analogously, LNPs comprising lipidoid or lipid-mimetic materials have exhibited potent siRNA-mediated silencing efficacy in rodents and non-human primates [133,134].

Small interfering RNA (siRNA) therapeutics possess the ability to selectively identify and degrade target mRNAs, thereby enabling the precise regulation of gene expression. These agents can be swiftly designed utilizing well-characterized gene sequences. Furthermore, siRNAs can undergo chemical modifications to improve their therapeutic efficacy, indicating considerable promise for the treatment of rare diseases, cancers, and other complex medical conditions. Nonetheless, the application of siRNA drugs is impeded by several challenges, including their vulnerability to cellular membrane barriers, complications associated with drug delivery, the potential for off-target effects, the possible disruption of normal gene expression, and elevated production costs [135]. These factors collectively limit the broader implementation of siRNA-based therapies.

In recent years, siRNA drugs have demonstrated therapeutic promise in areas, such as cancer treatment, viral infections [27], neurological treatment [38], and tumor therapy. In the context of primary hyperoxaluria, the implementation of nedosiran [41] provides a solution to this issue. In 2018, the first siRNA drug approved by the FDA, patisiran [29], was introduced, and by 2019, another siRNA drug, givosiran [32], for the treatment of acute hepatic porphyria (AHP), was also granted FDA approval. Inclisiran [34] received its initial approval from the European Union in December 2020 for the management of adults diagnosed with primary hypercholesterolemia, encompassing both heterozygous familial and non-familial forms, as well as mixed dyslipidemia, to be used in conjunction with dietary modifications. This rapid development underscores the immense potential of siRNA as a therapeutic modality. The advancement of siRNA technology is occurring at a swift pace and demonstrates considerable promise for future developments.

## 6. MicroRNAs (miRNAs)

MicroRNAs (miRNAs) are approximately 22 nucleotides in length, and their mode of action is analogous to that of small interfering RNAs (siRNAs). Initially, miRNAs are transcribed into primary microRNAs (pri-miRNAs) through the action of RNA polymerase II. Furthermore, pri-miRNAs undergo additional cleavage by Drosha enzymes during the transcription process, resulting in the formation of stem-loop precursors within the nucleus. Subsequently, in the cytoplasm [136], the stem-loop precursor is processed by the Dicer enzyme, leading to the production of double-stranded RNA. One of these strands associates with the Agonaute2 (AGO2) protease to create the miRNA-induced silencing complex (miRISC), which plays a crucial role in the subsequent degradation process. miRISC is capable of binding to mRNAs by complementing the sequences of target RNAs following their synthesis. The seed region of miRNAs consists of 2-7 nucleotides, which is crucial for identifying targets [137]. When the target mRNA exhibits complementarity to the central region of the miRNA, the AGO2 protein within the miRNA-induced silencing complex (miRISC) will demonstrate endonuclease activity, leading to the cleavage of the target mRNA [138] In general, mammalian miRNAs and their corresponding target mRNAs exhibit incomplete complementarity (Figure 4C). Consequently, miRNAs do not facilitate the direct cleavage of Argonaute 2 (AGO2); rather, their primary mechanisms of action involve the inhibition of translation and the promotion of mRNA degradation.

To enhance the stability of miRNAs in vivo and protect them from degradation by intracellular nucleases, it is common practice to chemically modify miRNA glycoconjugates. Substituents, including 2′-O-methyl and 2′-O-methoxyethyl groups, are incorporated at the 2′ position of miRNAs. The addition of these substituents have the potential to modify the conformation of the sugar ring, thereby enhancing the stability of the miRNAs against nuclease degradation [139]. Furthermore, incorporating sulfur atoms into the phosphodiester bonds of miRNAs can chemically alter them, reducing their hydrophilicity and enhancing their resistance to degradation by nucleases [140].

While chemical modification may improve the stability of miRNA to some degree, it does not fully address the challenges associated with the low efficiency of the cellular uptake of miRNA. Consequently, the advancement of delivery technologies is essential for the effective utilization of miRNA. To improve the efficiency of delivery and the uptake rate of miRNA, researchers have devised both viral and non-viral vectors. Nevertheless, viral vectors encounter significant obstacles, including elevated immunogenicity and limited loading capacity, which positions non-viral vectors as the preferred option for delivery [141]. Liposomes present several advantages, including straightforward preparation, safety, and non-toxicity [142]. The application of cationic or neutral liposomes as nanocarriers for the delivery of siRNA and miRNA has been the subject of extensive investigation, with some formulations advancing to clinical trial stages. A range of ionizable lipids has been synthesized, notably the ionizable cationic lipid DLin-MC3-DMA, which has been effectively employed in the siRNA therapeutic patisiran [16].

MicroRNA (miRNA) therapeutics possess the capacity to concurrently modulate multiple target genes, thereby presenting novel strategies for addressing diseases that involve multiple genetic factors. The conserved sequences inherent to miRNAs facilitate the development of broad-spectrum pharmacological agents, potentially lowering research and development expenditures and functioning as biomarkers for the early detection of diseases. Nonetheless, several challenges impede their clinical implementation, including the intricate nature of their mechanisms of action, the lack of targeted delivery systems, issues related to stability and susceptibility to degradation, and the insufficient advancement of large-scale production methodologies [143].

As research in the life sciences continues to expand, miRNAs have emerged as significant contributors to the understanding of cancer [144], metabolic disorders, viral infections, and various other domains. The initial investigation identifying miRNAs in plasma was published in 2008 [145], subsequently leading to the discovery of miRNAs in various other bodily fluids [146]. As of the present, research on miRNAs remains ongoing. Advances in sequencing technologies and gene editing techniques have substantially facilitated the progress of miRNA studies, indicating a favorable trajectory in this field.

## 7. Aptamer RNA

The term aptamer, derived from the Latin word “aptus” (meaning suitable) and the Greek word “meros” (meaning partial), refers to a single -stranded oligonucleotide or oligodeoxynucleotide with a short sequence length that can form a certain three-dimensional structure. It can specifically bind to target molecules, including metal ions, organic small-molecule compounds, nucleic acids, proteins, etc., and has functions similar to antibodies [147] (Figure 4D). The structures of aptamers include stem loops [148], internal loops, pseudoknots [149,150,151], kissing complexes [152,153], hairpin structures [154], G-quadruplex structures [155,156], etc. The binding affinity between aptamers and target molecules mainly includes aromatic ring stacking, electrostatic interactions, and hydrogen bonding interactions [157]. Based on these structures, in the environment of buffer salt ions, aptamers tend to fold and form more complex and specific three-dimensional spatial conformations, which provide favorable conditions for the high-affinity and high-specificity recognition and binding of aptamers to targets, such as heavy metals, pesticides, antibiotics, and biotoxins. Overall, the mechanism and mode of ligand target recognition resemble antibody–antigen immune binding, yet they possess distinct features compared to antibodies, rendering it a unique “chemical antibody”.

Aptamer drugs also face the obstacle problem of NADs, such as poor transmembrane ability and stability. The phosphate backbone of oligonucleotides endows them with strong hydrophilicity, which results in the poor transmembrane-entry ability of aptamers; oligonucleotides are easily degraded by the RNase widely present in blood, skin, and other organs, and have a short half-life in serum [158]. The above defects greatly limit the application of oligonucleotide drugs. To date, there is a lack of research reports addressing the delivery mechanisms of nucleic acid aptamer drugs. The majority of existing studies concentrate on the chemical modifications of nucleic acid aptamers subsequent to SELEX screening, a process referred to as post-SELEX modification. The chemical modification of nucleic acid aptamer drugs is primarily aimed at achieving three objectives: (1) enhancing the stability of nucleic acid aptamers to prolong their circulation time; (2) improving the transmembrane transport capabilities of nucleic acid aptamers to increase their cellular uptake efficiency while minimizing the required drug concentration; and (3) augmenting the binding affinity to the intended target. Various modifications have been extensively employed, including 2′-methoxynucleosides (2′-OMe) [159,160], 2′-fluorinated nucleosides (2′-F) [161,162,163], locked nucleic acids (LNAs) [164], 2′-fluorinated nucleosides, acyclic nucleosides (UNA) [165], L-nucleosides, heteronucleosides (D/LisoT) [166,167,168,169], and deoxyinosine (2′-dI) [170], among others. Research indicates that modifications, such as 2′-OMe, 2′-F, and 2′-NH_2_, significantly enhance the resistance of RNA aptamers to nuclease degradation [171]. However, the 2′-NH_2_ modification has become less prevalent due to inefficiencies in coupling during solid-phase synthesis and a propensity for adopting the C2′-internal (‘DNA-like’) glycan ring conformation [172].

To date, there is a lack of research reports addressing the delivery mechanisms of nucleic acid aptamer drugs. Liposomes and lipid nanoparticles (LNPs) are characterized by high encapsulation rates, enhanced transfection efficiencies, and minimal toxicity and immunogenicity [173]. Lipid complexes and lipid polycomplexes (LPPs) offer enhanced encapsulation and protection for aptamer RNA, while microbial LPP nanoplatforms are designed to specifically target dendritic cells, thereby activating T cell immune responses [174]. Despite the high specificity of the aptamer-mediated delivery system, there remain challenges related to attachment stability and endosomal escape efficiency [175]. Exosomes are noted for their biocompatibility and ability to traverse physiological barriers; however, they are limited by low preparation and loading efficiencies. Conversely, N-acetylgalactosamine (GalNAc)-conjugated delivery systems demonstrate effective targeting of the liver for the treatment of related diseases, although their application range is more restricted [176]. Future research on aptamer RNA delivery systems is expected to concentrate on optimizing the performance of existing systems, developing novel delivery mechanisms, and exploring the mechanisms of action and metabolism of aptamer RNA. Such investigations are anticipated to yield significant advancements in disease treatment.

Utilizing SELEX technology, RNA aptamer nucleic acid therapeutics can proficiently isolate aptamers from random nucleic acid libraries [177]. These aptamers exhibit the capability to target a diverse array of molecules with high affinity and specificity, thereby facilitating precise modulation of the functional activity of target molecules for purposes of disease diagnosis and targeted therapy. This methodology presents considerable potential for the treatment of cancer. Nevertheless, RNA aptamers are vulnerable to degradation by nucleases in vivo, and research concerning delivery systems remains in its nascent stages. Furthermore, the complexity and cost associated with the production process, along with the potential for immunogenicity, pose additional challenges that may restrict their clinical applicability.

The clinical advancement of aptamers for diagnostic applications and targeted drug delivery is presently trailing behind that of other NABTs. Although a number of aptamers have progressed to clinical trial phases, Macugen [20] remains the sole aptamer that has been successfully commercialized and implemented in clinical practice. Avacincaptad pegol [42] has recently received approval for the treatment of adult geographic atrophy associated with age-related macular degeneration. The primary challenges hindering the development of aptamers, in contrast to other small molecule pharmaceuticals, include an insufficient understanding of the pharmacokinetic characteristics of aptamers and the high costs associated with modification techniques.

## 8. Messenger RNA (mRNA)

mRNA therapy is a novel approach for delivering therapeutic proteins and peptides to cells by introducing messenger RNA into them [178]. This method avoids the risk of genomic integration and can be efficiently transfected into non-dividing cells, providing prolonged effects and enabling the expression of intracellular proteins. One of the major challenges in mRNA therapy is its high immunogenicity, which triggers an inflammatory response. To address this, the chemical modification of nucleosides in mRNA has emerged as a key technology to regulate immunogenicity.

The chemical modification of mRNA is mainly reflected in the synthesis process of IVT-mRNA (in vitro transcription-mRNA) [179]. Studies by Karikó et al. have shown that the chemical modification of this process can weaken the interaction between innate immune receptors and mRNA, significantly improving the stability and efficacy of mRNA [180]. IVT mRNA modified by m6A and s2U (2-Thiouridine) can inhibit the activation of TLR3, while IVT mRNA modified by m5C (5-Methylcytidine), m5U (5-Methyluridine), s2U (2-Thiouridine), m6A (N6-Methyladenosine), and ψ (pseudouridine) can inhibit the activation of TLR7 and TLR8. Currently, m5C and ψ are the most important mRNA-modification methods, which can significantly improve the translation efficiency of mRNA and have been widely used.

Because mRNA displays a negative charge and has a larger molecular size, it enters cells at a slower rate when it comes into play, and the compatibility with the cell membrane is low. Therefore, the delivery system is also essential for mRNA drugs. The delivery system for mRNA drugs is similar to that of other NADs, and can be divided into viral vectors and non-viral vectors [181]. Among them, lipid nanoparticles are the most widely used delivery vehicle and still play an important role in the delivery of mRNA [182]. Therapeutic mRNA can be assembled into LNP–mRNA complexes with polyethylene glycol-lipid, cholesterol, auxiliary lipids, and ionizable lipids through various processes, and then, these complexes can be precisely targeted to cells for transfer, which has recently been widely applied in mRNA drug research [183]. Contemporary mRNA vaccines employ biodegradable lipids, including SM-102 in Moderna’s mRNA-1273 vaccine and ALC-0315 in BioNTech’s BNT162b2 vaccine [184].

mRNA nucleic acid therapeutics are characterized by their unique ability to convey genetic information directly into cells, facilitating the production of specific proteins for the treatment of genetic disorders. The adaptability in their design during the development phase, coupled with the rapid synthesis capabilities once a target has been identified, significantly shortens the development timeline and has been instrumental in the creation of the COVID-19 vaccine [185]. Nevertheless, these therapeutics encounter several obstacles: they exhibit instability, are susceptible to degradation by nucleases, necessitate precise delivery mechanisms, suffer from underdeveloped technologies for large-scale production, incur high costs, and may elicit an immune response, all of which impede their broader implementation.

In recent years, as researchers’ studies on mRNA drugs have increased, the advantages of mRNA have gradually become apparent. The mRNA technology has achieved significant breakthroughs in the development of COVID-19 [36,43] vaccines and has therefore received widespread attention worldwide. The FDA-approved mResvia (Moderna, Cambridge, MA, USA) in May 2024, an mRNA vaccine targeting RSV-associated respiratory illness in adults aged ≥60 years, works by encoding the prefusion RSV F protein and has demonstrated efficacy rates of up to 80.9% in preventing symptomatic, laboratory-confirmed RSV-associated lower respiratory tract disease in clinical trials [45].

With the continuous progress of technology, the effectiveness and safety of mRNA vaccines and therapies will be further improved. One major drawback is their instability, as mRNA molecules are prone to degradation by RNAses, necessitating sophisticated delivery systems to ensure stability and effective cellular uptake. Additionally, mRNA vaccines can elicit strong immune responses, potentially leading to inflammation, local irritation, or allergic reactions [186]. Understanding and mitigating the immune reactivity associated with mRNA vaccines, especially in sensitive populations, is critical for ensuring their safety and efficacy. Streamlining large-scale production processes is also essential to reduce costs and increase efficiency, making mRNA vaccines more accessible globally.

## 9. CRISPR-Cas9 Gene Editing System

The CRISPR-Cas9 system [187] is a highly precise gene editing tool that consists of the following components: (1) Cas9 protein with DNase activity; (2) crRNA (CRISPR RNA) generated by CRISPR transcription; (3) tracrRNA (trans-activating CRISPR RNA), which complements crRNA and participates in trans-activation. The intrinsic adaptability of the CRISPR-Cas system reduces the likelihood of unintended consequences and allows a single crRNA to successfully target several closely related viral entities [22]. When operational, crRNA recognizes a specific site on the target DNA and binds to tracrRNA to form a stable complex. This complex induces Cas9 protein to target a specific gene location, where it exerts its endonuclease effect and cuts to form a DSB (double-strand break). In eukaryotic organisms, the cut gene site initiates two repair mechanisms: HDR (Homology Directed Repair) and NHEJ (Non-Homologous End Joining Repair). In normal circumstances, the body typically maintains the coding sequence of the target gene during HDR. However, in the presence of a homologous site provided by the donor plasmid, it can also insert foreign gene fragments through homologous recombination. The HDR pathway enables precise editing of the DNA sequence targeted by Cas9. The error-prone NHEJ pathway tends to involve base deletions or insertions or even large segments of deletions or inversions during repair, leading to the premature termination of encoded genes or production of proteins with incorrect sequences. Despite some bacteria lacking an effective NHEJ pathway, Cas9 can be specifically introduced into bacterial genomes, and CRISPR-Cas9 enables the editing of bacterial genomes. Therefore, leveraging the principles of DNA damage repair allows for achieving DNA deletions and insertions. It is beneficial to the study of gene functions [188].

Although not strictly an NAD in the traditional sense, CRISPR-Cas9 gene editing technology uses the DNA enzyme Cas9 directed by RNA, so it can also be considered a nucleic acid drug therapy. Exagamglogene autotemcel (Casgevy™), a CRISPR-Cas9-edited autologous CD34+ cell therapy promoting high fetal hemoglobin production, was approved by UK regulators on November 2023 (and later by USA and EU) for treating transfusion-dependent β-thalassemia and sickle cell disease in eligible patients aged ≥12 [189]. In recent years, CRISPR-Cas9 has rapidly developed and been applied across various eukaryotic cells, viruses, and even some bacteria, providing novel ideas and means into research on gene functions, disease resistance breeding [190], disease treatment [191] and so on.

The CRISPR-Cas9 gene editing system is capable of precisely targeting and cleaving specific DNA sequences guided by RNA, thereby facilitating accurate gene modifications. This technology has diverse applications, encompassing genetic alterations in both animal and plant species, as well as therapeutic interventions for cancer [192]. Its operational simplicity and cost-effectiveness further enhance its appeal. Nonetheless, there are significant concerns regarding off-target effects, wherein the system may unintentionally disrupt non-target genes. Furthermore, germline editing presents safety risks and ethical dilemmas, primarily due to the potential implications of modifying human genetic material.

Despite the revolutionary advancements CRISPR-Cas9 systems have brought to gene editing, a significant challenge limiting its broad application lies in its potential for off-target effects, where Cas9 may inadvertently target and cleave unintended genomic sites due to similarities between the sgRNA and non-target DNA sequences, leading to genomic instability. To address this issue, researchers are actively exploring diverse strategies, including enhancing the cleavage specificity of nucleases, shortening the functional activity duration of Cas9, employing more precise Cas9 variants, such as SaCas9, and modifying Cas proteins and gRNAs to reduce off-target effects while maintaining effective targeting [193,194,195]. The CRISPR/Cas technology provides the capability to utilize messenger RNAs (mRNAs) that encode Cas proteins, in addition to the direct application of Cas proteins themselves. Alternatively, a singular plasmid can be utilized to encode both Cas proteins and guide RNAs (gRNAs), which are subsequently processed into active gRNAs and Cas nucleases within the cell through transcription and translation mechanisms [196]. Recent research has indicated that the in vivo delivery of Cas9 mRNA and single guide RNA (sgRNA) via lipid nanoparticles featuring disulfide bonds (BAMEA-O16B) can achieve editing efficiencies nearing 80% [197]. However, despite these promising developments in mitigating off-target risks, the refinement and optimization of CRISPR therapy remain ongoing research areas. Future studies will continue to focus on developing safer and more efficient gene-editing tools, broadening the horizon for their medical applications.

## 10. Clinical Applications of Nucleic Acid Drugs

In theory, NADs hold the potential to treat any disease by selecting the correct nucleotide sequence on the target gene. Distinct from conventional pharmacological interventions, these modalities demonstrate sustained therapeutic effects attributable to their distinct molecular characteristics and biological stability. A growing number of NABT candidates have transitioned from preclinical development to regulatory approval across global clinical trial phases. Several types of NADs, including ASOs, aptamers, siRNAs, and mRNA vaccines, have been utilized to treat rare genetic diseases, cancer, ophthalmic diseases, cardiovascular diseases, and infectious diseases.

In clinical practice, NADs for rare genetic disorders have achieved significant clinical translation. Specifically, antisense oligonucleotides, including isotersen [198] and eplontersen [199], along with siRNA agents, like patisiran [132] and vutrisiran [38], mediate mRNA silencing to prevent the pathological accumulation of misfolded transthyretin (TTR) aggregates in cardiac and neural tissues, demonstrating therapeutic efficacy in transthyretin amyloidosis. Duchenne muscular dystrophy is a rare inherited muscular disease caused by mutations in the dystrophin gene, characterized by progressive neuromuscular degeneration culminating in life-threatening cardiopulmonary dysfunction. Besides the initially approved ASOs, which are all containing PMO modifications, such as casimersen [200], eteplirsen [201], golodirsen [30], and viltolarsen [33], novel phosphorodiamidate morpholino oligomers (PPMO) demonstrating enhanced tissue specificity have emerged [202]. Notably, Sarepta’s TP-5051 and PepGen’s PCN-ED051 [203] have progressed to Phase II trials following promising early clinical data. Amyotrophic lateral sclerosis is a fatal neurodegenerative disease demonstrating both sporadic (90%) and familial (10%) etiologies with well-characterized genetic determinants [204,205]. Gene-specific NADs targeting these molecular pathways have shown clinical potential, exemplified by the approved agent tofersen [206] and the Phase III investigational compound ulefnersen [207]. Primary hyperoxaluria type 1 is an autosomal recessive disorder resulting in recurrent nephrolithiasis and the irreversible deterioration of renal function. The siRNA therapeutic lumasiran [208] represents the first FDA-approved NAD for this condition, with nedosiran [41] currently under clinical evaluation as a novel oxalate synthesis inhibitor.

Beyond rare genetic disorders, nucleic acid therapeutics have demonstrated substantial advancements in oncology applications. The siRNA therapeutic cotsiranib [209], which specifically inhibits the TGF-β signaling pathway through RNA interference, has exhibited encouraging clinical outcomes. The NCT04669808 trial evaluating intratumoral administration achieved complete pathological responses in all participants, advancing to Phase II clinical evaluation. In neoplastic immunotherapy, mRNA-based platforms have reached critical milestones, with the IGV-001 vaccine [210] demonstrating favorable pharmacokinetic profiles and dose-dependent immunogenicity. The mRNA-4157/pembrolizumab combination regimen has advanced to Phase III development. NCT05933577 and NCT06077760 have also entered Phase III clinical trials [211] and have become the frontrunners for the approval of a class of mRNA cancer vaccines.

In ophthalmologic and cardiovascular therapeutics, nucleic acid modalities continue to achieve clinical breakthroughs. The FGF2-targeting RNA aptamer RBM-007 [212] has entered a Phase II evaluation for ocular indications. SYL1801 [213], an NRARP-directed siRNA formulated for topical ocular delivery, maintains optimal safety profiles in Phase II trials for neovascular AMD. Tiwanisiran (SYL1001) [214], targeting dry eye syndrome pathophysiology, is undergoing Phase III validation. The USH2A mutation-specific agent QR-421a [215] demonstrates potential in reversing inherited retinal degeneration. Cardiovascular applications show particular promise with inclisiran, the pioneering siRNA therapy approved for dyslipidemia management [216,217], while the ORION trial series (NCT03397121, NCT03399370, NCT03400800) confirms durable efficacy in Phase III investigations.

Furthermore, nucleic acid therapeutics have demonstrated unprecedented advancements in combating infectious pathogens, particularly through mRNA vaccine platforms. The global health crisis instigated by SARS-CoV-2 coronavirus infections has catalyzed revolutionary progress in RNA-based immunization technologies. These next-generation vaccines employ the precision targeting of viral spike protein epitopes critical for host cell recognition, effectively inhibiting viral internalization mechanisms while eliciting robust neutralizing antibody responses [218,219]. The first batch of the COVID-19 vaccine mRNA-1273, which entered clinical trials, successfully prepared the first samples in just 42 days, demonstrating the unique advantage of the short development cycle of nucleic acid drugs. Phase III clinical trial results showed a 94.1% prevention effectiveness and 100% prevention effectiveness against severe COVID-19 [220]. Hepatitis B virus (HBV) is also a significant virus affecting global health. Bepirovisen [221], an ASO drug targeting all HBV RNAs, effectively inhibits viral replication and has immunostimulatory activity, potentially contributing to the permanent clearance of HBV virus in the blood. Additionally, several other nucleic acid drugs targeting HBV are in Phase III clinical trials, with the earliest potential availability in 2026, including NCT05630807 and NCT05630820 [221]. Several siRNA drugs, including GSK-5637608 [222], VIR-2218 [223], and ARB-270729 [224], have entered Phase II clinical trials and shown promising HBV-inhibition capabilities, with results awaited.

## 11. Frontiers and Prospects

The field of NABTs has garnered significant interest within the biomedical community, demonstrating substantial advancements in both investigative research and clinical applications. NABTs offer several unparalleled advantages over conventional pharmaceuticals. They provide precision medicine by selectively targeting specific genes, leading to a more targeted therapeutic approach. These agents can persistently exert their effects within cells, thereby reducing the frequency of dosing. In terms of safety, their specificity for particular molecular targets generally results in a lower incidence of toxic side effects. Additionally, the abbreviated development timeline for NABTs facilitates a quicker route to market, addressing urgent clinical needs.

While numerous NADs exhibit robust preclinical efficacy, their clinical translation remains constrained by regulatory approvals. A critical barrier in therapeutic development centers on systemic biocompatibility challenges. Although the molecular attributes of NADs are well-characterized, immune activation risks and carrier-related cytotoxic effects from delivery vehicles (e.g., lipid nanoparticles) introduce significant safety hurdles [225]. Illustratively, preclinical evaluations of PPMO revealed dose-dependent nephrotoxicity in rodent models when conjugated with CPPs [226]. Similarly, cationic polymeric vectors, like PLL and PEI, have demonstrated pro-apoptotic and inflammatory responses in vivo [227].

The pharmacokinetic profiles and off-target effects represent additional translational bottlenecks. Emerging formulation strategies have diversified NAD complexes through structural engineering, wherein chemical modifications, delivery modalities, and administration routes critically govern absorption–distribution–metabolism–excretion (ADME) dynamics and therapeutic indices [228,229]. For example, ASOs carry dose-limiting risks of hepatorenal toxicity due to tissue accumulation, necessitating rigorous ADME profiling during clinical development [230].

Looking ahead, the future of NABTs will be focused on addressing these challenges. Key areas of development will include the optimization of delivery systems to enhance cellular uptake, innovative drug design to improve efficacy and reduce immunogenicity. The synergistic assembly of tailored oligonucleotide therapeutics, precision-engineered targeting moieties, and biomimetic delivery architectures forms the cornerstone of developing patient-specific genetic interventions that address unmet medical demands. For successful translation into mainstream clinical practice, nucleic-acid-based modalities must concurrently achieve manufacturing scalability, implement stringent quality assurance protocols, and establish cold-chain-compliant distribution networks.

## 12. Conclusions

The rapid evolution of NABTs has ushered in a transformative era in precision medicine, offering unprecedented opportunities to address previously intractable genetic, oncologic, and infectious diseases. Innovations in chemical modifications, delivery systems, and gene-editing technologies have significantly enhanced the stability, specificity, and efficacy of nucleic acid drugs. Despite these advancements, challenges such as systemic delivery inefficiencies, off-target effects, immunogenicity, and scalability for widespread clinical adoption remain critical barriers. Future research must prioritize the development of next-generation delivery platforms, refined chemical architectures, and comprehensive safety profiling to unlock the full therapeutic potential of NABTs. By bridging interdisciplinary innovations and addressing translational bottlenecks, nucleic acid therapeutics are poised to redefine treatment paradigms across diverse medical disciplines, ultimately delivering on their promise of personalized and curative medicine.

## Data Availability

Not applicable.
